# Supplementary material for: Suicide-Related Single Nucleotide Polymorphisms, rs4918918 and rs10903034: Association with Dementia in Older Adults
Source: Genes (Basel). 2022 Nov 21;13(11):2174. doi: 10.3390/genes13112174 (PMC9690628; doi:10.3390/genes13112174)
Supplement: Supplementary file 1 [file genes-13-02174-s001.zip › genes-1997524-supplementary.pdf]

Supplementary materials

Table S1: Analysis of the association of allele frequency with the study groups (dementia compared to controls). Age was used as a covariate.

Model	Genotype	OR (95% CI)	P-value	AIC	BIC
		rs429358			
**Codominant**	T/T	1.00	**0.001**	263.5	277.6
	T/C	**2.23 (1.16-4.31)**			
	C/C	**16.34 (1.80-148.49)**			
**Dominant**	T/T	1.00	**0.002**	265.7	276.3
	T/C-C/C	**2.68 (1.42-5.06)**			
**Recessive**	T/T-T/C	1.00	**0.0047**	267.3	277.9
	C/C	**12.57 (1.41-112.41)**			
		rs7412			
**Codominant**	C/C	1.00	**0.02**	272.4	286.6
	C/T	**0.31 (0.12-0.81)**			
	T/T	NA (0.00-NA)			
**Dominant**	C/C	1.00	**0.024**	273.1	283.8
	C/T-T/T	**0.36 (0.14-0.91)**			
**Recessive**	C/C-C/T	1.00	0.22	276.7	287.3
	T/T	NA (0.00-NA)			
		rs9475195			
**Codominant**	T/T	1.00	0.35	217.7	230.8
	C/T	1.33 (0.63-2.80)			
	C/C	1.97 (0.79-4.94)			
**Dominant**	T/T	1.00	0.24	216.5	226.3
	C/T-C/C	1.51 (0.75-3.01)			
**Recessive**	T/T-C/T	1.00	0.21	216.3	226.1
	C/C	1.68 (0.74-3.82)			
		rs7982251			
**Codominant**	T/T	1.00	0.52	252.5	266.2
	C/T	1.43 (0.67-3.07)			

	C/C	2.40 (0.25-23.24)			
**Dominant**	T/T	1.00	0.29	250.7	260.9
	C/T-C/C	1.49 (0.71-3.12)			
**Recessive**	T/T-C/T	1.00	0.5	251.3	261.6
	C/C	2.20 (0.23-21.04)			
		rs2834789			
**Codominant**	T/T	1.00	0.43	232.5	245.8
	C/T	1.12 (0.56-2.23)			
	C/C	1.94 (0.71-5.32)			
**Dominant**	T/T	1.00	0.46	231.6	241.6
	C/T-C/C	1.28 (0.67-2.43)			
**Recessive**	T/T-C/T	1.00	0.21	230.6	240.6
	C/C	1.83 (0.71-4.74)			
		rs358592			
**Codominant**	T/T	1.00	0.45	220.2	233.5
	C/T	0.64 (0.32-1.28)			
	C/C	0.89 (0.26-3.05)			
**Dominant**	T/T	1.00	0.25	218.5	228.4
	C/T-C/C	0.68 (0.35-1.31)			
**Recessive**	T/T-C/T	1.00	0.88	219.8	229.8
	C/C	1.10 (0.33-3.60)			
		rs4918918			
**Codominant**	C/C	1.00	0.079	232.4	245.7
	C/T	0.55 (0.28-1.10)			
	T/T	0.38 (0.14-1.03)			
**Dominant**	C/C	1.00	**0.033**	230.9	240.9
	C/T-T/T	**0.50 (0.26-0.95)**			
**Recessive**	C/C-C/T	1.00	0.14	233.3	243.3
	T/T	0.50 (0.19-1.27)			
		rs3781878			

**Codominant**	G/G	1.00	0.19	202.8	215.7
	A/G	1.79 (0.86-3.74)			
	A/A	2.48 (0.57-10.87)			
**Dominant**	G/G	1.00	0.076	201	210.7
	A/G-A/A	1.88 (0.93-3.80)			
**Recessive**	G/G-A/G	1.00	0.35	203.2	213
	A/A	1.95 (0.46-8.18)			
		rs10903034			
**Codominant**	T/T	1.00	0.43	232.2	245.6
	C/T	1.57 (0.77-3.18)			
	C/C	1.50 (0.59-3.84)			
**Dominant**	T/T	1.00	0.19	230.2	240.3
	C/T-C/C	1.55 (0.80-3.01)			
**Recessive**	T/T-C/T	1.00	0.71	231.8	241.8
	C/C	1.18 (0.50-2.76)			
		rs165774			
**Codominant**	G/G	1.00	0.26	224	237.2
	A/G	1.10 (0.56-2.19)			
	A/A	2.86 (0.80-10.27)			
**Dominant**	G/G	1.00	0.45	224.1	234
	A/G-A/A	1.29 (0.67-2.46)			
**Recessive**	G/G-A/G	1.00	0.11	222	232
	A/A	2.75 (0.79-9.53)			
		rs16841143			
**Codominant**	G/G	1.00	0.81	225.6	238.8
	A/G	0.79 (0.34-1.84)			
	A/A	0.63 (0.06-6.91)			
**Dominant**	G/G	1.00	0.53	223.6	233.5
	A/G-A/A	0.77 (0.34-1.74)			
**Recessive**	G/G-A/G	1.00	0.73	223.9	233.8

	A/A	0.66 (0.06-7.18)			
		rs11833579			
**Codominant**	G/G	1.00	0.3	226.8	240.2
	A/G	0.67 (0.34-1.33)			
	A/A	0.40 (0.10-1.72)			
**Dominant**	G/G	1.00	0.16	225.3	235.3
	A/G-A/A	0.63 (0.33-1.21)			
**Recessive**	G/G-A/G	1.00	0.3	226.2	236.2
	A/A	0.48 (0.12-1.97)			
		rs10898553			
**Codominant**	C/C	1.00	0.5	194.5	207
	C/T	1.21 (0.53-2.77)			
	T/T	1.82 (0.66-5.01)			
**Dominant**	C/C	1.00	0.44	193.3	202.6
	C/T-T/T	1.36 (0.62-2.99)			
**Recessive**	C/C-C/T	1.00	0.28	192.7	202.1
	T/T	1.60 (0.68-3.78)			
		rs7296262			
**Codominant**	T/T	1.00	0.85	230.9	244.1
	C/T	1.15 (0.56-2.37)			
	C/C	1.31 (0.51-3.37)			
**Dominant**	T/T	1.00	0.62	228.9	238.9
	C/T-C/C	1.19 (0.60-2.36)			
**Recessive**	T/T-C/T	1.00	0.67	229	239
	C/C	1.20 (0.52-2.77)			
		rs3806263			
**Codominant**	G/G	1.00	0.9	227.3	240.5
	A/G	0.87 (0.44-1.72)			
	A/A	1.08 (0.30-3.91)			
**Dominant**	G/G	1.00	0.75	225.4	235.3

	A/G-A/A	0.90 (0.47-1.72)			
**Recessive**	G/G-A/G	1.00	0.83	225.5	235.4
	A/A	1.15 (0.33-4.01)			
		rs2462021			
**Codominant**	T/T	1.00	0.83	225.5	238.7
	C/T	0.90 (0.45-1.82)			
	C/C	1.35 (0.38-4.88)			
**Dominant**	T/T	1.00	0.92	223.9	233.8
	C/T-C/C	0.97 (0.50-1.88)			
**Recessive**	T/T-C/T	1.00	0.58	223.6	233.5
	C/C	1.41 (0.41-4.91)			

AIC - Akaike information criterion; BIC - Bayesian information criterion; MMSE - Mini-Mental State Examination; OR - Odds ratio; P-value < 0.05 was considered as significance;

Table S2. Analysis of the association of genotypes with the MMSE score. Age was used as a covariate.

Model	Genotype	Mean MMSE	Difference (95% CI)	P-value	AIC	BIC
			rs429358			
**Codominant**	T/T	24.41 (0.63)	0.00	**0.038**	1395.4	1412.1
	T/C	21.84 (1.24)	**-2.60 (-4.69 - -0.51)**			
	C/C	21.5 (2.7)	-3.09 (-8.67 - 2.49)			
**Dominant**	T/T	24.41 (0.63)	0.00	**0.011**	1393.4	1406.8
	T/C-C/C	21.81 (1.15)	**-2.65 (-4.66 - -0.63)**			
**Recessive**	T/T-T/C	23.69 (0.57)	0.00	0.41	1399.4	1412.7
	C/C	21.5 (2.7)	-2.36 (-7.97 - 3.25)			
			rs7412			
**Codominant**	C/C	23.33 (0.62)	0.00	**0.014**	1411.8	1428.6
	C/T	26.59 (0.97)	**3.33 (0.60 - 6.07)**			
	T/T	11 (0)	-11.30 (-24.61 - 2.00)			
**Dominant**	C/C	23.33 (0.62)	0.00	**0.044**	1414.4	1427.8
	C/T-T/T	26.04 (1.09)	**2.81 (0.09 - 5.52)**			
**Recessive**	C/C-C/T	23.75 (0.56)	0.00	0.089	1415.5	1428.9
	T/T	11 (0)	-11.73 (-25.18 - 1.72)			
			rs9475195			
**Codominant**	T/T	23.17 (1.05)	0.00	0.22	1092.1	1107.4
	C/T	22.85 (0.97)	0.26 (-2.29 - 2.80)			
	C/C	20.14 (1.91)	-2.51 (-5.84 - 0.81)			
**Dominant**	T/T	23.17 (1.05)	0.00	0.68	1093	1105.3
	C/T-C/C	22.1 (0.88)	-0.51 (-2.91 - 1.88)			
**Recessive**	T/T-C/T	22.99 (0.71)	0.00	0.084	1090.1	1102.4
	C/C	20.14 (1.91)	-2.66 (-5.65 - 0.34)			
			rs7982251			
**Codominant**	T/T	22.96 (0.7)	0.00	0.92	1259.3	1275.4
	C/T	23.32 (1.43)	-0.26 (-2.85 - 2.33)			
	C/C	23.25 (3.33)	-1.35 (-8.48 - 5.77)			

**Dominant**	T/T	22.96 (0.7)	0.00	0.77	1257.4	1270.3
	C/T-C/C	23.32 (1.32)	-0.36 (-2.85 - 2.12)			
**Recessive**	T/T-C/T	23.03 (0.63)	0.00	0.72	1257.4	1270.2
	C/C	23.25 (3.33)	-1.30 (-8.38 - 5.79)			
			rs2834789			
**Codominant**	T/T	22.71 (0.98)	0.00	0.95	1157.5	1173.1
	C/T	22.23 (1)	0.11 (-2.21 - 2.44)			
	C/C	23.06 (2.05)	0.61 (-3.22 - 4.44)			
**Dominant**	T/T	22.71 (0.98)	0.00	0.86	1155.5	1168
	C/T-C/C	22.38 (0.9)	0.20 (-2.00 - 2.41)			
**Recessive**	T/T-C/T	22.47 (0.7)	0.00	0.77	1155.5	1168
	C/C	23.06 (2.05)	0.55 (-3.10 - 4.20)			
			rs358592			
**Codominant**	T/T	22.76 (1.04)	0.00	0.42	1119.4	1135
	C/T	23.6 (0.89)	1.01 (-1.26 - 3.28)			
	C/C	21 (2.6)	-1.32 (-5.15 - 2.51)			
**Dominant**	T/T	22.76 (1.04)	0.00	0.58	1118.9	1131.3
	C/T-C/C	23.15 (0.86)	0.61 (-1.56 - 2.79)			
**Recessive**	T/T-C/T	23.19 (0.68)	0.00	0.32	1118.2	1130.6
	C/C	21 (2.6)	-1.84 (-5.48 - 1.80)			
			rs4918918			
**Codominant**	C/C	21.95 (0.97)	0.00	0.76	1135	1150.5
	C/T	23.02 (1.08)	0.70 (-1.75 - 3.15)			
	T/T	23.71 (1.81)	1.12 (-2.29 - 4.53)			
**Dominant**	C/C	21.95 (0.97)	0.00	0.48	1133	1145.5
	C/T-T/T	23.2 (0.92)	0.81 (-1.45 - 3.08)			
**Recessive**	C/C-C/T	22.45 (0.72)	0.00	0.63	1133.3	1145.7
	T/T	23.71 (1.81)	0.79 (-2.41 - 3.99)			
			rs3781878			
**Codominant**	G/G	22.82 (0.86)	0.00	0.23	1029	1044.1

	A/G	21.38 (1.31)	-2.11 (-4.59 - 0.37)			
	A/A	20.88 (3.6)	-2.00 (-7.31 - 3.30)			
**Dominant**	G/G	22.82 (0.86)	0.00	0.086	1027	1039.1
	A/G-A/A	21.32 (1.22)	-2.09 (-4.47 - 0.28)			
**Recessive**	G/G-A/G	22.26 (0.73)	0.00	0.66	1029.8	1041.9
	A/A	20.88 (3.6)	-1.19 (-6.44 - 4.06)			
			rs10903034			
**Codominant**	T/T	24.89 (0.82)	0.00	**0.024**	1142.3	1157.9
	C/T	21.64 (1.06)	**-2.80 (-5.07 - -0.53)**			
	C/C	21.07 (1.66)	**-3.39 (-6.40 - -0.39)**			
**Dominant**	T/T	24.89 (0.82)	0.00	**0.0068**	1140.4	1153
	C/T-C/C	21.48 (0.89)	**-2.96 (-5.08 - -0.84)**			
**Recessive**	T/T-C/T	23.11 (0.7)	0.00	0.19	1146.2	1158.7
	C/C	21.07 (1.66)	-1.86 (-4.64 - 0.91)			
			rs165774			
**Codominant**	G/G	22.9 (0.89)	0.00	0.81	1118.5	1134
	A/G	22.04 (1.09)	-0.34 (-2.62 - 1.95)			
	A/A	25.12 (2.95)	1.40 (-3.81 - 6.60)			
**Dominant**	G/G	22.9 (0.89)	0.00	0.9	1116.9	1129.3
	A/G-A/A	22.37 (1.02)	-0.15 (-2.36 - 2.06)			
**Recessive**	G/G-A/G	22.53 (0.69)	0.00	0.56	1116.6	1129
	A/A	25.12 (2.95)	1.54 (-3.56 - 6.64)			
			rs16841143			
**Codominant**	G/G	22.11 (0.78)	0.00	0.7	1116.7	1132.1
	A/G	23.36 (1.52)	0.64 (-2.35 - 3.62)			
	A/A	28 (1.35)	2.82 (-4.48 - 10.12)			
**Dominant**	G/G	22.11 (0.78)	0.00	0.53	1115	1127.4
	A/G-A/A	23.94 (1.36)	0.90 (-1.92 - 3.73)			
**Recessive**	G/G-A/G	22.33 (0.69)	0.00	0.47	1114.8	1127.2
	A/A	28 (1.35)	2.70 (-4.56 - 9.96)			

			rs11833579			
**Codominant**	G/G	21.92 (0.93)	0.00	0.49	1146.3	1161.8
	A/G	22.85 (1.03)	-0.34 (-2.67 - 2.00)			
	A/A	24.78 (2.28)	2.81 (-2.23 - 7.84)			
**Dominant**	G/G	21.92 (0.93)	0.00	0.97	1145.7	1158.2
	A/G-A/A	23.08 (0.95)	0.04 (-2.22 - 2.30)			
**Recessive**	G/G-A/G	22.32 (0.69)	0.00	0.24	1144.3	1156.8
	A/A	24.78 (2.28)	2.95 (-1.98 - 7.87)			
			rs10898553			
**Codominant**	C/C	20.67 (1.59)	0.00	0.5	929.5	944
	C/T	22.61 (0.93)	1.69 (-1.19 - 4.58)			
	T/T	22.04 (1.77)	1.52 (-2.24 - 5.28)			
**Dominant**	C/C	20.67 (1.59)	0.00	0.24	927.5	939.1
	C/T-T/T	22.47 (0.82)	1.65 (-1.10 - 4.39)			
**Recessive**	C/C-C/T	21.93 (0.82)	0.00	0.8	928.8	940.4
	T/T	22.04 (1.77)	0.42 (-2.85 - 3.69)			
			rs7296262			
**Codominant**	T/T	22.77 (1.1)	0.00	0.6	1119	1134.4
	C/T	22.44 (1)	-0.94 (-3.50 - 1.63)			
	C/C	20.87 (1.64)	-1.63 (-4.93 - 1.68)			
**Dominant**	T/T	22.77 (1.1)	0.00	0.36	1117.2	1129.5
	C/T-C/C	22.02 (0.85)	-1.12 (-3.54 - 1.29)			
**Recessive**	T/T-C/T	22.57 (0.74)	0.00	0.48	1117.5	1129.9
	C/C	20.87 (1.64)	-1.06 (-3.97 - 1.85)			
			rs3806263			
**Codominant**	G/G	22.77 (0.92)	0.00	0.49	1096.7	1112
	A/G	22.48 (1.05)	-0.48 (-2.86 - 1.90)			
	A/A	20.15 (3.15)	-2.59 (-6.86 - 1.68)			
**Dominant**	G/G	22.77 (0.92)	0.00	0.47	1095.6	1107.9
	A/G-A/A	22.09 (1.02)	-0.84 (-3.10 - 1.43)			

**Recessive**	G/G-A/G	22.65 (0.69)	0.00	0.26	1094.8	1107.1
	A/A	20.15 (3.15)	-2.38 (-6.52 - 1.75)			
			rs2462021			
**Codominant**	T/T	23.7 (0.84)	0.00	0.31	1104.8	1120.2
	C/T	21.9 (1.07)	0.01 (-2.35 - 2.36)			
	C/C	17.5 (4.45)	-4.04 (-9.26 - 1.19)			
**Dominant**	T/T	23.7 (0.84)	0.00	0.7	1105.1	1117.4
	C/T-C/C	21.41 (1.07)	-0.44 (-2.73 - 1.84)			
**Recessive**	T/T-C/T	22.97 (0.66)	0.00	0.12	1102.8	1115.1
	C/C	17.5 (4.45)	-4.04 (-9.15 - 1.07)			

AIC - Akaike information criterion; BIC - Bayesian information criterion; MMSE - Mini-Mental State Examination; OR - Odds ratio; P-value < 0.05 was considered as significance;
